# Self-reported sexually transmitted infections and associated risk factors among female university students

**DOI:** 10.48101/ujms.v129.10943

**Published:** 2024-09-30

**Authors:** Sofie Smeds, Cerisa Obern, Inger Sundström Poromaa, Johan Westerbergh, Tanja Tydén, Frida Gyllenberg

**Affiliations:** a Department of Women’s and Children’s Health, Uppsala University, Uppsala, Sweden; b Uppsala Clinical Research Center, Uppsala University, Uppsala, Sweden; c Department of General Practice and Primary Health Care, University of Helsinki and Helsinki University Hospital, Helsinki, Finland

**Keywords:** Sexually transmitted diseases, sexual behavior, unsafe sex, sexual health, young adult

## Abstract

**Background:**

The spread of sexually transmitted infections (STIs) is an ongoing public health challenge, and awareness of risk factors is essential for designing effective preventive interventions. This study aimed to assess self-reported STI occurrences and identify risk factors and sexual behaviors associated with STIs among female university students.

**Methods:**

This is a cross-sectional, online questionnaire study, including 384 female university students seeking contraceptive counseling at a gynecology clinic in Uppsala, Sweden, and reporting having had sex. Associated risk factors and behaviors were assessed by comparing those who reported STIs and those who did not.

**Results:**

The mean age of participants was 22.8 years. Seventy-eight (20%) had contracted at least one STI, with seven (9%) experiencing multiple infections. Seventy-three (94%) reported first-date sexual activity without a condom among STI experienced. Chlamydia trachomatis was the most common STI pathogen (68% of all infections), followed by Herpes simplex virus (18%) and Mycoplasma genitalium (13%). Behavioral factors associated with self-reported STIs were first-date sexual activity without a condom, not using condom at first intercourse, younger age at first intercourse, a higher number of sexual partners overall and in the last 12 months, experience of anal sex, dating app usage, and regretting sexual activity after substance use (*P* < 0.003 for all).

**Conclusions:**

Condom use was low among the respondents, and STIs were common regardless of the high level of education in this group. Contraceptive counseling needs to highlight the importance of condom use in addition to contraceptive efficacy. It is also essential to consider the specific risk factors and behaviors prevalent among young adults to reduce the spread of STIs.

## Introduction

Sexually transmitted infections (STIs) are a common problem worldwide and present various challenges regarding diagnostics, treatment, and prevention in high-, middle-, and low-income countries. Most STIs are not life-threatening, but they can still lead to complications such as pelvic inflammatory disease and increase the risk of ectopic pregnancy, infertility, and fetal and neonatal morbidity (1). Due to differences in the urogenital anatomy between sexes, women are particularly susceptible to complications caused by STIs (2).

University students can be considered especially vulnerable to contracting an STI, as partying, including binge drinking and recreational drug use, is a common and normalized behavior in campus culture (3, 4). Several studies have shown associations between binge drinking (5, 6), smoking (7), drug use (8), and sexual risk behavior, such as not using a condom (9, 10). At this age, many students are also looking for a partner, and it is common to date as well as engage in one-night stands. This has become even easier as dating apps have become more popular among university students, facilitating contact with a possible life partner or sexual partner. Previous studies have found that dating app use or meeting sexual partners online is associated with more sexual risk-taking for both sexes. This includes one-night stands, multiple sexual partners, and sex without condom use (11–14), all of which increase the risk of contracting an STI. Worryingly, many individuals consider themselves to be at low risk even though they engage in many high-risk sexual behaviors (15–17).

Studies about sexual behavior and the use of contraceptives among female university students in Uppsala have been conducted regularly since 1989 (18–23). The aim of the current study was to investigate the occurrence of STIs among female university students reporting having had sex and visiting a gynecology clinic in Uppsala, Sweden, and to describe current sexual behaviors associated with having had an STI.

## Materials and methods

Uppsala is a Swedish university city with about 50,000 students. Swedish-speaking women who visited an outpatient gynecology clinic for contraceptive counseling were asked to participate. Data collection took place between February and June 2023. Previously, only university students could visit this clinic, which was designated as the student healthcare clinic. From 2014 onward, it became open to all women.

When attending the clinic, women were invited to participate and received information about the study along with a QR link to the survey. The survey was conducted via REDCap (24) and could be answered on one’s smartphone. Participation was voluntary and anonymous. The goal was to collect 500 responses, but ultimately 599 were collected, and of these, 384 were university students reporting having had sex.

No ethical permission was needed since the study participants were anonymous, and no sensitive personal information could be linked to any individual (advisory opinion, Swedish Ethical Review Authority, dnr 219-04587).

Since this was a follow-up study, the questionnaire was mostly identical to those used in previous studies (21–23). A few new questions have been added over the years in response to contemporary phenomena. This resulted in a total of 52 questions, most of which were multiple-choice and some open-ended. The present study focused on 24 of the 52 questions. The complete questionnaire is available upon request.

Statistical analyses were conducted in IBM SPSS Statistics (version 29.0.2.0) and R (version 4.2.3). Continuous and discrete variables were analyzed using T-test for normally distributed data and Mann-Whitney U-test for skewed data. Categorical variables were analyzed using Pearson’s chi-square test and Fisher’s exact test when there were too few observations for Pearson’s chi-square test. Proportions of STIs are presented with 95% confidence intervals (CIs) calculated using Clopper-Pearsons exact CIs. Due to the exploratory nature of the study and testing multiple characteristics, we set the level of statistical significance at 0.003 in accordance with the Bonferroni correction method (0.05/20 = 0.0025 ≈ 0.003).

## Results

In total, 599 persons answered the questionnaire. As this study focuses on university students, those who stated that they worked, went to upper secondary school, or did not answer the question about occupation were excluded from further analyses. In addition, we only included participants reporting having had sex. Thus, 384 women studying at the university remained, and the background characteristics of these are presented in Table 1. The students’ internal response rates varied between 97 and 100% for multiple-choice questions and 83 and 100% for the open-ended questions analyzed in this study.

**Table 1:** Background characteristics of study participants (*n* = 384).

	*n*	%	Mean ± SD
Mean age (years)			22.8 ± 2.8
Country of birth			
Sweden	355	92.4	
Other	27	7.0	
Studying			
Uppsala University	321	83.6	
Swedish University of Agricultural Sciences	40	10.4	
Other university	27	7.0	
Smoke			
Sometimes	41	10.7	
Daily	3	0.8	
Brown snuff			
Sometimes	7	1.8	
Daily	4	1.0	
White snuff			
Sometimes	78	20.3	
Daily	66	17.2	
Currently in stable relationship	205	53.4	
Time in current relationship (years)			2.5 ± 2.5
Sexual orientation			
Heterosexual	302	78.6	
Homosexual	4	1.0	
Bisexual	72	18.8	
Other	4	1.0	

Out of the 384 study participants, 78 (20%, 95% CI 16.4 – 24.7%) had experienced at least one STI, and seven (9%, 95% CI 3.7 – 17.6%) of them reported a history of more than one STI. Chlamydia trachomatis was the most commonly occurring STI; see Table 2 for more information.

**Table 2:** Self-reported STI pathogens among female university students (*n* = 78).

STI	*n*	%^a^
Chlamydia trachomatis	53	67.9
Herpes simplex virus (genital)	14	17.9
Mycoplasma genitalium	10	12.8
Human papilloma virus	4	5.1
Neisseria gonorrhoea	2	2.6

a The percentage of STI pathogens adds up to 106.3% because seven females reported more than one pathogen

Table 3 presents the results from the comparison between individuals who have experienced an STI and those who have not. As shown, age at first intercourse, number of sexual partners, not using condom at first intercourse, first-date sexual activity without a condom, experience of anal sex, dating app usage, and regret of sexual activity after substance use were associated with the experience of an STI (*P* < 0.003).

**Table 3:** Characteristics and sexual behavior related to STI status among female university students.

	Have had an STI		*p*-value^b^
	Yes (*n* = 78)	No (*n* = 306)	
Participant age	23.5 ± 3.4	22.7 ± 2.6	0.016^1^
Age at first intercourse (years), mean ± SD	16.1 ± 2.0	17.2 ± 2.2	< 0.001^1^
Currently in stable relationship	35 (44.9)	170 (55.6)	0.081^2^
Total number of sexual partners, mean ± SD	21.1 ± 18.0	7.6 ± 8.2	< 0.001^3^
Number of sexual partners during past 12 months, mean ± SD	4.1 ± 3.7	2.2 ± 2.1	< 0.001^3^
Condom usage			
First intercourse, *n* (%)	40 (51,^3^)	214 (69.9)	0.002^2^
First intercourse with latest partner, *n* (%)	24 (30.8)	137 (44.8)	0.025^2^
Latest intercourse, *n* (%)	12 (15.4)	72 (23.5)	0.120^2^
First-date sexual activity without condom, *n* (%)	73 (93.6)	176 (57.5)	< 0.001^2^
Experience of anal sex, *n* (%)	39 (50.0)	82 (26.8)	< 0.001^2^
Condom use during anal sex, *n* (%)^a^	18 (46.2)	44 (53.7)	0.107^2^
Always, *n* (%)	5 (12.8)	17 (20.7)	
Sometimes, *n* (%)	13 (33.3)	27 (32.9)	
Dating app usage, *n* (%)	51 (65.4)	123 (40.2)	< 0.001^4^
Sexual partners through app, mean ± SD	5.4 ± 6.5	3.8 ± 4.8	0.019^3^
HPV-vaccinated, *n* (%)	67 (85.9)	272 (88.9)	0.463^2^
Experience of induced abortion, *n* (%)	8 (10.3)	11 (3.6)	0.034^4^
Used emergency contraception, *n* (%)	54 (69.2)	168 (54.9)	0.022^2^
Number of times used, mean ± SD	2.2 ± 1.6	1.9 ± 1.5	0.123^3^
Regretted sexual activity after substance use, *n* (%)	52 (66.7)	92 (30.1)	< 0.001^4^
Alcohol, *n* (%)	44 (56.4)	91 (29.7)	
Alcohol and drugs, *n* (%)	8 (10.3)	1 (0.3)	
Snuff user, *n* (%)	39 (50.0)	107 (35.0)	0.041^2^
Daily, *n* (%)	20 (25.6)	49 (16.0)	
Sometimes, *n* (%)	19 (24.4)	58 (19.0)	
Smoker, *n* (%)	14 (17.9)	30 (9.8)	0.056^4^
Daily, *n* (%)	0 (0.0)	3 (1.0)	
Sometimes, *n* (%)	14 (17.9)	27 (8.8)	

a Among those who reported having had anal sex (*n* = 121)

b Comparison of those who have had an STI with those who have not

1 T-test,

2 Pearson’s chi-square test,

3 Mann-Whitney U-test,

4 Fisher’s exact test

## Discussion

Of the respondents, 20% had experienced an STI, and 9% of these had experienced multiple STIs. Both of these estimates were nominally lower than in the previous survey from 2014, when 26% reported one STI and 17% of these reported multiple STIs (18). The decline in the number of infections is in line with Swedish national statistics on Chlamydia trachomatis infections, which report on more cases in 2014 as compared to 2022 (36,125 vs 32,808) (25). The occurrence of Neisseria gonorrhoeae, on the other hand, has increased from 1,336 to 3,356 reported cases during the same period (26). This is also reflected among the university students in our sample and is worrying in view of the spread of antibiotic-resistant strains (1). Reporting of Mycoplasma genitalium was low in this study despite it being estimated to be almost as common as Chlamydia trachomatis (27).

It is possible that the tendency toward a reduction in STI occurrence can be partly explained by the COVID-19 pandemic. In Sweden, the Public Health Agency issued recommendations such as limiting close contacts and keeping a distance from others to limit the spread of the virus (28). These measures affected the possibility of meeting new people, and may have influenced opportunities for meeting potential sexual partners during the pandemic. Another partial explanation for the slightly lower STI rates might be a later sexual debut, which could have an impact at the populational level. Additionally, the age at first intercourse was slightly higher in the current study than in 2014 (17.0 vs 16.7), a finding also reported previously (29).

A significant difference in the number of sexual partners was observed between women with and without experience of an STI, with more sexual partners in the STI group, in line with previous studies (30, 31). The age at first intercourse differed by just over a year between the groups, with the STI group being younger, also in line with prior research (31).

Dating app usage and meeting sexual partners online have been associated with sexual risk behavior (13, 14) and are supported by this study. This indicates that it is easy to get in touch with potential sexual partners through dating apps, which often promote casual sexual encounters through their design. Young adults are a target audience for these apps, and in Sweden, 45% of Generation Z singles have used dating apps (32). Among them, many are university students, highlighting this particular risk factor for STIs in this demographic.

Among those who reported experiencing an STI, it was more than twice as common to report having ‘regretted sexual activity after substance use’ compared to those without STI experience. Finally, considering the risk of contracting an STI, it is worrying that more than half of all students and almost all the students in the STI group have experienced first-date sexual activity without a condom. As recruitment took place at a gynecology clinic, our study does not capture those using only condom and hence not needing an appointment to obtain a prescription. This might affect the low rate of condom use. However, condom use is always recommended with new partners despite the use of other contraceptives. It cannot be excluded that the low numbers are also affected by impaired judgment due to alcohol consumption, as one-night stands often occur after a night out. However, non-use of condom was not statistically significantly associated with STIs in all situations. This suggests that other risk behaviors besides sex without a condom are more important for STI risk, as one can behave responsibly, for example, by getting tested before having sex with a new partner and therefore not using a condom.

### Strengths and limitations

With its cross-sectional design, this study only provides information on associations between known variables. Nonetheless, it is a good-sized sample from a clearly defined population and offers valuable insights into factors associated with STIs among university students. We also set a conservative significance level, safeguarding against false positives and overinterpretation of the results. Additionally, the high internal response rate further strengthens the study.

All recruited participants attended the same gynecological clinic, which may indicate a certain sociodemographic group and cause selection bias. On the other hand, the clinic’s central location and popularity among students facilitated effective recruitment. Condom-only users rarely visit a clinic for contraceptive counseling, which may also contribute to selection bias. Participation in the study was anonymous, which we considered important to minimize the risk of social desirability bias, as most of the questions were on sensitive topics. A potential downside of anonymity is that we could not examine the representativeness of the sample, and there is a risk of non-response bias if those who declined participation represent a specific group. Furthermore, all the data collected were self-reported retrospectively, making recall bias likely to some extent since a person’s ability to accurately recall past events deteriorates with time. The reliability of self-reported diagnoses depends on many factors, including the type of condition, question design, and participant understanding. With self-reported diagnoses, it is impossible to know if the person truly had the disease and had it verified by healthcare professionals or merely self-diagnosed. Particularly in the case of STIs, which carry stigma, accurate reporting may be further compromised. Altogether, this might contribute to an underestimated STI occurrence.

### Implications and further research

This study’s findings contribute to the existing literature on STIs and sexual risk behaviors, which is crucial for designing preventive measures and promoting sexual health among young adults. Despite efforts to prevent STIs, poor condom use remains a persistent problem, especially among dating app users and those under the influence of alcohol. While this study does not delve into the reasons behind the lack of condom use, further research in this area is essential to identify specific barriers and develop targeted interventions. Future studies should continue to monitor STI occurrence beyond the COVID-19 pandemic, as the digital age and widespread use of dating apps may change people’s sexual habits and behaviors.

## Conclusions

The identified risk factors and behaviors for contracting an STI included younger age at first intercourse, a greater number of sexual partners, not using condom at first intercourse, first-date sexual activity without a condom, experience of anal sex, dating app usage, and regret of sexual activity after substance use. Despite the high level of education in this group, there was a worrying occurrence of STIs and a lack of condom use, especially under the influence of alcohol. These risk factors and behaviors should be considered when planning prevention efforts in this demographic group, and continuous promotion of condom use should be prioritized, especially in a risk context.
